# Perioperative risk factors for acute kidney injury after off-pump coronary artery bypass grafting: a retrospective study

**DOI:** 10.1186/s40981-017-0125-2

**Published:** 2017-10-04

**Authors:** Yuta Kumada, Kenji Yoshitani, Yusuke Shimabara, Yoshihiko Ohnishi

**Affiliations:** 1 Department of Anesthesiology, National Cerebral and Cardiovascular Center, 5-7-1, Fujishirodai, Suita, Osaka 565-8565 Japan; 2 Department of Cardiac Surgery, National Cerebral and Cardiovascular Center, 5-7-1, Fujishirodai, Suita, Osaka 565-8565 Japan

**Keywords:** AKI, Acute kidney injury, Off-pump coronary artery bypass grafting, Furosemide

## Abstract

**Background:**

Acute kidney injury (AKI) after cardiac surgery is associated with increased morbidity and mortality. Although morbidity of AKI after off-pump coronary artery bypass grafting (OPCAB) has been investigated, little is known about risk factors for AKI after OPCAB. To identify risk factors for AKI, we examined the association between perioperative variables and AKI after OPCAB.

**Findings:**

We reviewed the medical records of consecutive adult patients who underwent isolated OPCAB between January 2010 and February 2013 in a single institute, retrospectively. The primary outcome was the incidence of AKI evaluated using Acute Kidney Injury Network classifications during the first 48 h postoperatively. We investigated preoperative and intraoperative variables, including hemodynamic parameters, as potential risk factors for AKI. The relationship between candidates of AKI and incidence of AKI was examined by multivariate logistic regression analysis.

A total of 298 patients were enrolled in this study. Acute kidney injury occurred in 47 patients (15.7%). Multivariate logistic regression analysis showed that intraoperative furosemide administration (odds ratio [OR], 5.163; 95% confidence interval, 2.171 to 12.185; *P* < 0.001] and diabetes mellitus (OR, 1.954; 95% confidence interval, 1.004 to 3.880; *P* = 0.049) were significantly associated with AKI.

**Conclusions:**

Intraoperative furosemide administration and diabetes mellitus were significantly associated with AKI in patients who had received OPCAB.

## Introduction

Acute kidney injury (AKI) is a highly prevalent and serious complication after cardiac surgery. Depending on the various definitions of AKI, 5 to 40% of patients who have undergone cardiac surgery developed AKI, and 1 to 5% of patients required dialysis [1–6]. The mortality rate of patients who required dialysis after cardiac surgery reached 50 to 70%, whereas the subclinical increase in the serum creatinine level was associated with a more than 3-fold mortality rate [7, 8]. To predict this serious complication, numerous studies have identified several risk factors for AKI after cardiac surgery; however, most of them targeted cardiac surgery with cardiopulmonary bypass (CPB), such as coronary artery bypass grafting (CABG) [3–6, 9].

Off-pump CABG (OPCAB) has been expected to have a lower incidence of AKI than on-pump CABG (ONCAB), as CPB has been proven to cause an inflammatory response, lack of pulsatile flow, hemodilution, atheroembolism, hemolysis, low output syndrome, and global hypoperfusion, which may lead to postoperative complications. However, the benefit of OPCAB for AKI remains controversial. Several studies have reported that patients with OPCAB have a lower incidence of AKI than those with ONCAB [10–14]; yet, other studies could not find any significant difference between OPCAB and ONCAB [15–18]. Possible explanations for a high incidence of AKI in OPCAB include hemodynamically induced renal ischemia, atheroembolism from side clamping, and a technical difficulty requiring heart displacement. Instead of using CPB, OPCAB requires heart displacement during anastomosis of grafts, potentially resulting in intraoperative hemodynamic instability and global hypoperfusion [19]. However, few studies have focused on the relationship between intraoperative hemodynamic instability and the incidence of AKI after OPCAB [20–24].

In this retrospective study, we examined the association between AKI after OPCAB with perioperative variables, including intraoperative parameters related to hemodynamic instability, such as the cardiac index and lactate levels.

## Methods

This study was approved by the institutional review board of our center in June 2013 (Number: M25-025). We retrospectively analyzed the data of consecutive adult patients who underwent isolated OPCAB between January 2010 and February 2013 at the center. We excluded patients with end-stage kidney disease with an estimated glomerular filtration rate (eGFR) < 15 mL/min/1.73 m^2^ or requiring dialysis, a history of previous cardiac surgery, congenital heart disease, and minimally invasive cardiac surgery. We focused on patients with AKI after OPCAB as a primary outcome. The incidence of AKI was diagnosed until 48 h postoperatively, and the staging of AKI was evaluated until 7 days postoperatively, according to the criteria proposed by Acute Kidney Injury Network (AKIN) classifications [25] as AKIN stage 1: increased creatinine level > 50% or 0.3 mg/dL from baseline, or urine output < 0.5 mL/kg/h continuing more than 6 h; AKIN stage 2: increased creatinine level by > 100%, or urine output < 0.5 mL/kg/h over 12 h; and AKIN stage 3: increased creatinine level by > 200% or serum creatinine level > 4 mg/dL with an acute increase of > 0.5 mg/dL, new-onset renal replacement therapy, urine output < 0.3 mL/kg/h over 24 h, or anuria over 12 h. We examined preoperative and intraoperative variables for associations with AKI after OPCAB. Categories of preoperative risk factors of AKI included age, body mass index, preoperative comorbidities, laboratory data, emergent surgery, coronary angiography within 7 days, and perioperative intra-aortic balloon pumping (IABP). For intraoperative variables, we investigated the use of diuretics, vasopressors, cardiac index (CI), base excess (BE), serum lactate levels, and hemoglobin levels during surgery.

## Anesthesia technique

All patients underwent general anesthesia. We routinely use a direct arterial blood catheter, transesophageal echocardiography, and a pulmonary artery catheter with a continuous CI measurement. When transient circulatory failure and global hypoperfusion occur during manipulation of the heart or anastomosis, we usually maintain systemic perfusion pressure by tilting the surgical table and administering intravenous fluids followed by the administration of noradrenaline as a first-choice vasopressor.

## Statistical analysis

Continuous variables are expressed as a mean ± standard deviation [SD]. Categorical variables are expressed as a number and percentage. Initially, all preoperative variables and intraoperative variables, except continuous variables in the arterial blood gas analysis during surgery, were divided into dichotomous data according to a clinically significant cut point. To identify which variables were associated with AKI, data were examined using the chi-square test or Fisher’s exact test for dichotomous data, or univariate logistic regression analysis for intraoperative continuous data. Among the variables with a *P* value < 0.20 in univariate analysis, we selected covariates in multivariable logistic regression analysis considering known risk factors or relationships to hemodynamics during surgery to determine independent risk factors for AKI. To avoid overfitting of the model, we allowed a maximum of one variable per each 10 events. Model calibration was assessed using the Hosmer-Lemeshow goodness-of-fit test. A two-sided *P* value < 0.05 was considered statistically significant. All statistical analyses were performed using SPSS software, version 21.0 (IBM, Armonk, NY, USA).

## Results

Of 339 patients enrolled, 41 were excluded from the analysis, because the patients had end-stage kidney disease with an eGFR < 15 mL/min/1.73 m^2^, required dialysis (*n* = 23), or had a history of cardiac surgery (*n* = 10), minimally invasive cardiac surgery (*n* = 2), or OPCAB with another surgery (*n* = 6). Two hundred ninety-eight patients remained in this study. Approximately 16% of patients had AKI, and most of them were classified as stage 1 according to the AKIN criteria (Table 1).

**Table 1:** Postoperative AKI and perioperative creatinine value

Incidence of AKI^a^	47 (15.7%)
Stage 1	42
Stage 2	4
Stage 3	1
Preoperative serum creatinine level (mg/dL)	0.94 ± 0.34
Highest postoperative serum creatinine level (mg/dL)	1.07 ± 0.47

Data are presented as a number (%) or mean ± standard deviation

*AKI* acute kidney injury

^a^Eleven of 40 patients in stage 1 and half of patients in stage 2 met only urine criteria without the clinical increase in the serum creatinine level. One patient in stage 3 had anuria and received renal replacement therapy on postoperative day 2

Details related to the surgery and anesthesia are shown in Table 2. Most patients had 3-vessels coronary disease or left main coronary artery involvement; therefore, they underwent more than three grafts of anastomosis. All patients were operated through a median sternotomy incision, and the left internal thoracic artery was anastomosed to the left anterior descending coronary artery; this was subsequently followed by revascularization of the left circumflex artery and right coronary artery. Exposure of the grafting site and motionless surgical field required deep pericardial traction sutures and commercially available stabilizers. A coronary shunt was routinely inserted before graft anastomosis.

**Table 2:** Clinical features of the surgery and anesthesia (*n* = 298)

Operative time (min)	290 ± 55
Anesthesia time (min)	386 ± 61
Number of anastomoses	3.6 ± 0.9
Bypass area	
LAD	291 (100%)
LCX	255 (85.6%)
RCA	226 (75.8%)
Emergent surgery	61 (21.0%)
Maintenance of anesthesia	
Propofol	255 (85.6%)
Sevoflurane	44 (14.8%)
Noradrenaline	274 (91.9%)
Maximum dose (μg/kg/min)	0.11 ± 0.11
Dopamine	267 (89.6%)
Maximum dose (μg/kg/min)	2.4 ± 1.2
Other inotropes	31 (10.4%)

Data are presented as a mean ± standard deviation or number (%)

*LAD* left anterior descending artery, *LCX* left circumflex artery, *RCA* right coronary artery

Results of univariate analysis of preoperative demographic characteristics and intraoperative variables are shown in Table 3. The following three variables were significantly associated with AKI in univariate analysis (*P* < 0.05): diabetes mellitus, perioperative IABP, and intraoperative furosemide administration. Besides these three variables, preoperative hypoalbuminemia (albumin level < 4 g/dL), the highest intraoperative lactate level, and total urine output during surgery  were likely to increase the incidence of AKI (*P* < 0.20). However, since urine output itself was involved in the diagnosis of AKI after surgery from the end of surgery to 48 h after surgery, we thought intraoperative urine output as clearly inappropriate for a risk factor to be analyzed by multivariate analysis. We entered the five variables with a *P* value < 0.20 except total urine output  into multivariable logistic regression analysis, because five covariates were within permissible range to avoid overfitting the model based on occurrence of 47 outcomes in this study. Multivariable logistic regression analysis identified intraoperative furosemide administration and diabetes mellitus as independent risk factors for AKI (Table 4). The Hosmer-Lemeshow goodness-of-fit test showed an adequate performance of the predictive model (*P* = 0.914).

**Table 3:** Preoperative demographic characteristics and intraoperative variables in patients with or without AKI

Variable	AKI (*n* = 47)	No AKI (*n* = 251)	*P* value
Age > 75 years	8 (17.0%)	52 (20.7%)	0.5620
Female sex	10 (21.3%)	50 (19.9%)	0.8315
BMI > 25 kg/m^2^	20 (42.6%)	84 (33.5%)	0.2303
Comorbid disease			
Hypertension	45 (95.7%)	226 (90.0%)	0.2111
Hyperlipidemia	40 (85.1%)	216 (86.1%)	0.8637
Diabetes mellitus	29 (61.7%)	107 (42.6%)	0.0160
Ejection fraction < 35%	6 (12.8%)	22 (8.8%)	0.4131
Atrial fibrillation	2 (4.3%)	12 (4.8%)	1.0000
Peripheral vascular disease	9 (19.2%)	70 (27.9%)	0.2128
Cerebrovascular disease	9 (19.2%)	58 (23.1%)	0.5508
Preoperative laboratory data			
Hemoglobin level < 8 g/dL	0 (0%)	1 (0.4%)	1.0000
Albumin level < 4 g/dL	21 (44.7%)	85 (33.9%)	0.1551
eGFR mL/min/1.73 m^2^	62.6 ± 20.4	64.2 ± 17.5	0.5745
eGFR < 60 mL/min/1.73 m^2^	17 (36.1%)	95 (37.9%)	0.8274
Preoperative clinical condition			
CAG within 7 days	13 (27.7%)	62 (24.7%)	0.6680
Emergent operation	12 (25.5%)	49 (19.5%)	0.3487
Intra-aortic balloon pumping	12 (25.5%)	34 (13.6%)	0.0369
Intraoperative variable			
Lowest BE level	− 3.7 ± 2.0	− 3.4 ± 2.4	0.3491
Highest lactate level (mmol/L)	1.5 ± 0.6	1.3 ± 0.6	0.1807
Lowest hemoglobin level (g/dL)	8.3 ± 0.9	8.2 ± 0.8	0.8708
Lowest CI < 1.8 (L/min/m^2^)	18 (41.9%)	84 (40.4%)	0.8576
Furosemide administration	13 (27.7%)	15 (6.0%)	< 0.001
Carperitide administration	8 (17.0%)	28 (11.2%)	0.2574
Noradrenaline > 0.1 μg/kg/min	12 (25.5%)	84 (33.4%)	0.3126
Total urine output (ml)	1209 ± 948	1458 ± 873	0.0666

Data are presented as a mean ± standard deviation or number (%). Continuous data were assessed using univariate logistic regression analysis, and categorical data were examined using the chi-square test or Fisher’s exact test

*AKI* acute kidney injury, *BMI* body mass index, *eGFR* estimated glomerular filtration rate, *CAG* coronary angiography, *BE* base excess, *CI* cardiac index

**Table 4:** Results of multivariable logistic regression analysis for postoperative AKI

Covariate	Odds ratio	95% confidence interval	*P* value
Intraoperative furosemide administration	5.163	2.171 to 12.185	< 0.001
Diabetes mellitus	1.954	1.004 to 3.880	0.049
Preoperative intra-aortic balloon pumping	1.601	0.669 to 3.673	0.279
Intraoperative highest lactate level	1.168	0.697 to 1.850	0.5325
preoperative hypoalbuminemia (albumin level < 4 g/dL)	1.042	0.498 to 2.116	0.9106

*AKI* Acute kidney injury

We further reviewed the patients who were administered furosemide based on the above results. In all 28 patients with furosemide, only single injection, not continuous infusion, was used to administer furosemide, and the average dosage of furosemide was 10 ± 4.90 mg. Although this retrospective study could not clearly specify the reason why furosemide was used for oliguria, hyperkalemia, or hypervolemia during surgery, only four patients (14.2%) experienced continued oliguria during surgery (total urine output during surgery < 0.5 ml/kg/h). Since diuretics were usually used to treat oliguria, it was difficult to interpret the exact relationship between furosemide and postoperative AKI, whether the furosemide was either risk factor, or simply risk marker. Therefore, we also investigated the preoperative kidney function and intraoperative urine output in the patients given the furosemide or carperitide, the another diuretic commonly used for oliguria as well as furosemide (Table 5). The patients given either furosemide or carperitide demonstrated significantly smaller amount of urine output and lower preoperative eGFR than those without the diuretics. However, carperitide but furosemide did not increase the incidence of AKI, and intraoperative urine output did not increase the incidence of AKI in univariate analysis (Table 3), suggesting that furosemide itself might have any potential effect on the incidence of AKI.

**Table 5:** The patients demographics given the furosemide or carperitide during surgery.

Variable	Diuretics (+)	Diuretics (-)	*P* value
Furosemide administration	*n* = 28	*n* = 270	
Preoperative eGFR (mL/min/1.73 m^2^)	57.5 ± 21.1	64.6 ± 17.5	0.0457
Intraoperative urine output (ml)	763.3 ± 670.9	1487.5 ± 881.7	<0.001
Carperitide administration	*n* = 36	*n* = 262	
Preoperative eGFR (mL/min/1.73 m^2^)	45.4 ± 17.0	66.5 ± 16.5	<0.001
Intraoperative urine output (ml)	815.8 ± 794.1	1502.4 ± 870.0	<0.001

Data are presented as a mean ± standard deviation. Statistical analyses were performed by the unpaired Student’s *t*-test.

AKI = acute kidney injury; eGFR = estimated glomerular filtration rate.

## Discussion

In this retrospective study, multivariable logistic regression analysis identified intraoperative furosemide administration and diabetes mellitus as independent risk factors for AKI. Intraoperative hemodynamic parameters, CI, BE, lactate, and hemoglobin levels were not associated with AKI.

Diabetes mellitus is well known as a systemic disease, which frequently causes the diabetic nephropathy, neuropathy, retinopathy, and increased the cardiovascular events and mortality [26, 27]. Diabetic nephropathy, the leading cause of chronic kidney disease worldwide, is one of the most common complications in diabetic patient, and the diabetic patient is more likely to suffer from renal damage than those without diabetes, even if the nephropathy was not evident [28, 29]. Indeed, previous studies suggested that diabetes is an important risk factor for postoperative AKI after cardiac surgery [4, 5, 22, 30–32]. Recently, Hertzberg et al. conducted the large observational study including 36,106 patients with CABG, which suggested that type 2 diabetes significantly increased the incidence of postoperative AKI defined by AKIN criteria after CABG (OR 1.27; confidence interval 1.16–1.40) [30]. In the present study, diabetes mellitus was significantly associated with postoperative AKI after cardiac surgery (OR, 1.954; 95% confidence interval, 1.00–3.880; *P* = 0.049). This result was consistent with previous studies [4, 5, 22, 30–32].

We also identified the use of furosemide during surgery as an independent risk factor for postoperative AKI. Furosemide is a loop diuretic that inhibits the Na-K-2Cl cotransporter system in the luminal membrane of the ascending limb of the loop of Henle [33, 34]. Furosemide has been widely prescribed to maintain or increase urine output for both the risk and treatment of AKI, because it may be needed to maintain patency of renal tubules and prevent tubular obstruction and back-leak. In addition, furosemide may reduce renal tubular oxygen consumption by decreasing sodium reabsorption, which leads to less ischemic damage of the most vulnerable outer medullary tubular segments [35–38].

However, prior studies have failed to demonstrate a clinical benefit of furosemide in terms of mortality and renal recovery of AKI and suggested a possible harmful effect of furosemide when used to prevent or treat AKI. [37–45] A previous multicenter, observational study that enrolled 552 patients with AKI in intensive care units from the Program to Improve Care in Acute Renal Disease (PICARD) study group demonstrated that the use of furosemide was associated with a significant increase in the risk of death or non-recovery of renal function (odds ratio [OR], 1.77; 95% confidence interval, 1.14 to 2.76) [39]. A recent meta-analysis of 9 randomized, controlled trials of 849 patients that compared furosemide with a placebo to prevent or treat acute renal failure concluded that furosemide did not reduce in-hospital mortality (relative risk 1.11, 95% confidence interval 0.92 to 1.33), the requirement for dialysis (relative risk 0.99, 95% confidence interval 0.80 to 1.22), number of dialysis sessions required until recovery of renal function, proportion of patients remaining oliguric, or length of hospital stay [40]. Based on these evidence, The Kidney Disease Improving Global Outcomes Clinical Practice Guideline for Acute Kidney Injury proposed not using diuretics to prevent or treat AKI (1B recommendation), except in the management of volume overload (2C recommendation) [38].

Although most previous studies did not focus on the effect of furosemide on postoperative AKI, several studies have implied a deleterious effect of furosemide in cardiac operation [6, 44, 45]. Parolari et al. reported, based on a prospective, observational study of 3219 patients after cardiac surgery, that multivariable logistic regression analysis identified furosemide during CPB as an independent predictor for postoperative AKI [6]. Lassnigg et al. conducted a double-blind randomized, controlled trial of 126 patients with normal renal function undergoing elective cardiac surgery and found that a continuous infusion of furosemide was associated with a higher rate of renal impairment [44]. They suggested that the negative effect of furosemide was due to two possible theories: first, neurohumoral activation of the sympathetic and renin-angiotensin systems by furosemide could increase peripheral vascular resistance, left ventricular afterload, and cardiac work, thus mediating a decrease in cardiac output; and, second, the induction of maldistribution of renal blood flow with diversion of medullary perfusion by a decrease in cortical vascular resistance may promote tubular dysfunction. These previous findings support our results [6, 37–45]. We retrospectively examined recorded data at a single center, meaning that both causality and the mechanisms by which furosemide had a detrimental effect on renal function could not be analyzed in this study. However, a possible explanation is that an unnatural increasing of urine output may delay or mask the recognition of AKI, which would forfeit an opportunity for optimal treatment against the cause of AKI [39]. In addition, it has been proposed that furosemide may cause a decrease in the circulating volume and activate the neurohumoral response, such as the sympathetic nervous system and renin-angiotensin system, which could increase peripheral vascular resistance, left ventricular afterload, and decrease cardiac output [37, 44, 46–49].

Although we aimed to investigate whether intraoperative hemodynamic instability during OPCAB affected postoperative AKI, we were unable to identify anything regarding an association between intraoperative hemodynamic parameters and postoperative AKI. Intraoperative hemodynamic instability can cause the impairment of renal perfusion, resulting in postoperative AKI. A recent meta-analysis involving data of 4220 surgical patients in 20 studies examined whether perioperative hemodynamic optimization improved renal outcome and found that postoperative AKI was significantly reduced by perioperative hemodynamic optimization compared with that in the control group (OR 0.64; confidence interval 0.50 to 0.83; *P* = 0.0007) [50]. However, in our study, intraoperative hemodynamic instability was not associated with postoperative AKI. Our inability to detect the impact of intraoperative hemodynamic instability on renal function in this study may be due to the temporal nature of the data collected within our hemodynamic parameters. Although we investigated the lowest CI, lowest BE, and highest lactate levels intraoperatively, we did not review the duration of the unstable data, nor the quality of the data recovery.

There were several limitations in this study. First, the number of patients was small and derived from a single center. Even though many preoperative factors including age, anemia, hypoalbuminemia, DM, chronic kidney disease, chronic heart failure, and IABP have been reported as the independent risk factors for AKI in early studies, we could not identify the only two independent factors [4, 5, 9, 21, 22]. There may be possible selection bias and a lack of power. Second, data for the cardiac index were missing in about 19% of the study populations that included four patients with AKI. This may decrease the power of this study to evaluate this hemodynamic parameter as risk factors for AKI. Third, we did not analyze the intraoperative systemic blood pressure. Several previous observational studies reported that lower mean arterial pressure during surgery was involved in postoperative AKI [51, 52]. However, we could not collect the detailed data for arterial blood pressure due to the form of this retrospective survey. Additionally, we frequently altered the bed positioning to maintain hemodynamic condition during anastomosis, which readily affected the arterial blood pressure by changing the positional relation between introducer and right atrium. If the detailed data on arterial blood pressure was collected, the validity and reliability of the data might not have been guaranteed. Thus, as the index indirectly reflecting systemic perfusion pressure, we investigated the dose of vasopressors, which was not significantly associated with AKI in the present study. Finally, this study was a retrospective, observational study, and thus, causality between furosemide and AKI could not be established. It was possible that furosemide did not increase the incidence of AKI, as it was only used by patients who were likely to have renal impairment due to other means. However, whether furosemide was used against transient oliguria that might indicate postoperative AKI or not, urine output was recovered in most patients administered furosemide, and another diuretic, carperitide, did not affect the incidence of AKI. Thus, we could not deny that the use of furosemide itself had an adverse effect on postoperative AKI.

In conclusion, we found that intraoperative furosemide administration and diabetes mellitus were independently associated with AKI in patients undergoing OPCAB. However, our findings in this retrospective study represent only the possibility that furosemide during surgery affects postoperative AKI. Further prospective randomized trials are needed to investigate the effect of intraoperative furosemide administration on renal function, and whether intraoperative hemodynamic variables are associated with the incidence of AKI.

## Abbreviations

AKI: Acute kidney injury
AKIN: Acute Kidney Injury Network
BE: Base excess
CABG: Coronary artery bypass grafting
CAG: Coronary angiography
CI: Cardiac index
CPB: Cardiopulmonary bypass
eGFR: Estimated glomerular filtration rate
IABP: Intra-aortic balloon pumping
LAD: Left anterior descending artery
LCX: Left circumflex artery
ONCAB: On-pump CABG
OPCAB: Off-pump coronary artery bypass grafting
RCA: Right coronary artery
SD: Standard deviation

## References

[1] Hobson CE, Yavas S, Segal MS, Schold JD, Tribble CG, Layon AJ, Bihorac A (2009). Acute kidney injury is associated with increased long-term mortality after cardiothoracic surgery. Circulation.

[2] Rydén L, Sartipy U, Evans M, Holzmann MJ (2014). Acute kidney injury after coronary artery bypass grafting and long-term risk of end-stage renal disease. Circulation.

[3] Karkouti K, Wijeysundera DN, Yau TM, Callum JL, Cheng DC, Crowther M, Dupuis JY, Fremes SE, Kent B, Laflamme C, Lamy A, Legare JF, Mazer CD, McCluskey SA, Rubens FD, Sawchuk C, Beattie WS (2009). Acute kidney injury after cardiac surgery: focus on modifiable risk factors. Circulation.

[4] Rosner MH, Okusa MD (2006). Acute kidney injury associated with cardiac surgery. Clin J Am Soc Nephrol.

[5] Mangano CM, Diamondstone LS, Ramsay JG, Aggarwal A, Herskowitz A, Mangano DT (1998). Renal dysfunction after myocardial revascularization: risk factors, adverse outcomes, and hospital resource utilization. The Multicenter Study of Perioperative Ischemia Research Group. Ann Intern Med.

[6] Parolari A, Pesce LL, Pacini D, Mazzanti V, Salis S, Sciacovelli C, Rossi F, Alamanni F (2012). Monzino Research Group on Cardiac Surgery Outcomes. Risk factors for perioperative acute kidney injury after adult cardiac surgery: role of perioperative management. Ann Thorac Surg.

[7] Tolpin DA, Collard CD, Lee VV, Virani SS, Allison PM, Elayda MA, Pan W (2012). Subclinical changes in serum creatinine and mortality after coronary artery bypass grafting. J Thorac Cardiovasc Surg.

[8] Lassnigg A, Schmidlin D, Mouhieddine M, Bachmann LM, Druml W, Bauer PHM (2004). Minimal changes of serum creatinine predict prognosis in patients after cardiothoracic surgery: a prospective cohort study. J Am Soc Nephrol.

[9] Chertow GM, Lazarus JM, Christiansen CL, Cook EF, Hammermeister KE, Grover F, Daley J (1997). Preoperative renal risk stratification. Circulation.

[10] Di Mauro M, Gagliardi M, Iaco AL, Contini M, Bivona A, Bosco P, Gallina S, Calafiore AM (2007). Does off-pump coronary surgery reduce postoperative acute renal failure? The importance of preoperative renal function. Ann Thorac Surg.

[11] Nigwekar SU, Kandula P, Hix JK, Thakar CV (2009). Off-pump coronary artery bypass surgery and acute kidney injury: a meta-analysis of randomized and observational studies. Am J Kidney Dis.

[12] Seabra VF, Alobaidi S, Balk EM, Poon AH, Jaber BL (2010). Off-pump coronary artery bypass surgery and acute kidney injury: a meta-analysis of randomized controlled trials. Clin J Am Soc Nephrol.

[13] Lamy A, Devereaux PJ, Prabhakaran D, Taggart DP, Hu S, Paolasso E, Straka Z, Piegas LS, Akar AR, Jain AR, Noiseux N, Padmanabhan C, Bahamondes J-C, Novick RJ, Vaijyanath P, Reddy S, Tao L, Olavegogeascoechea PA, Chrolavicius B, Yusuf S, Phil D (2012). for the CORONARY Investigators. Off-pump or on-pump coronary-artery bypass grafting at 30 days. N Engl J Med.

[14] Chawla LS, Zhao Y, Lough FC, Schroeder E, Seneff MG, Brennan JM (2012). Off-pump versus on-pump coronary artery bypass grafting outcomes stratified by preoperative renal function. J Am Soc Nephrol.

[15] Shroyer AL, Grover FL, Hattler B, Collins JF, McDonald GO, Kozora E, Lucke JC, Baltz JH, Novitzky D, for the Veterans Affairs Randomized On/Off Bypass (ROOBY) Study Group (2009). On-pump versus off-pump coronary-artery bypass surgery. N Engl J Med.

[16] Diegeler A, Börgermann J, Kappert U, Breuer MDM, Böning MD, PhD A, Ursulescu MDA (2013). Off-pump versus on-pump coronary-artery bypass grafting in elderly patients. N Engl J Med.

[17] Reents W, Hilker M, Börgermann J, Albert M, Plötze K, Zacher M, Diegeler A, Boning A (2014). Acute kidney injury after on-pump or off-pump coronary artery bypass grafting in elderly patients. Ann Thorac Surg.

[18] Tang AT, Knott J, Nanson J, Hsu J, Haw MP, Ohri SK (2002). A prospective randomized study to evaluate the renoprotective action of beating heart coronary surgery in low risk patients. Eur J Cardiothorac Surg.

[19] Mathison M, Edgerton JR, Horswell JL, Akin JJ, Mack MJ (2000). Analysis of hemodynamic changes during beating heart surgical procedures. Ann Thorac Surg.

[20] Matsuda S, Fukui T, Shimizu J, Takao A, Takanashi S, Tomoike H (2013). Associations between preoperative anemia and outcomes after off-pump coronary artery bypass grafting. Ann Thorac Surg.

[21] Lee EH, Baek SH, Chin JH, Choi DK, Son HJ, Kim WJ, Hahm K-D, Sim J-Y, Choi I-C (2012). Preoperative hypoalbuminemia is a major risk factor for acute kidney injury following off-pump coronary artery bypass surgery. Intensive Care Med.

[22] Hong S, Youn Y-N, Yoo K-J (2010). Metabolic syndrome as a risk factor for postoperative kidney injury after off-pump coronary artery bypass surgery. Circ J.

[23] Lee EH, Chin JH, Joung KW, Choi D-K, Kim W-J, Lee J-B, Hahn K-D, Sim J-Y, Choi I-C (2013). Impact of the time of coronary angiography on acute kidney injury after elective off-pump coronary artery bypass surgery. Ann Thorac Surg.

[24] Yoo YC, Youn Y-N, Shim JK, Kim JC, Kim NY, Kwak YL (2010). Effects of renin-angiotensin system inhibitors on the occurrence of acute kidney injury following off-pump coronary artery bypass grafting. Circ J.

[25] Mehta RL, Kellum JA, Shah SV, Molitoris BA, Ronco C, Warnock DG, Levin A, Acute Kidney Injury Network (2007). Acute Kidney Injury Network: report of an initiative to improve outcomes in acute kidney injury. Crit Care.

[26] Reidy K, Kang HM, Hostetter T, Susztak K (2014). Molecular mechanisms of diabetic kidney disease. J Clin Invest.

[27] Gregg EW, Li Y, Wang J, Burrows NR, Ali MK, Rolka D, Williams DE, Geiss L (2014). Changes in Diabetes-Related Complications in the United States, 1990–2010. N Engl J Med.

[28] Adler AI, Stevens RJ, Manley SE, Bilous RW, Cull CA, Holman RR, UKPDS GROUP (2003). Development and progression of nephropathy in type 2 diabetes : The United Kingdom Prospective Diabetes Study (UKPDS64). Kidney Int.

[29] Ninomiya T, Perkovic V, Galan BED, Zoungas S, Pillai A, Jardine M, Patel A, Cass A, Neal B, Poulter N, Mogensen CE, Cooper M, Marre M, Williams B, Hamet P, Mancia G, Woodward M, Macmahon S, Chalmers J, ADVANCE Collaborative Group (2009). Albuminuria and kidney function independently predict cardiovascular and renal outcomes in diabetes. J Am Soc Nephrol.

[30] Hertzberg D, Sartipy U, Holzmann MJ, Stockholm S (2015). Type 1 and type 2 diabetes mellitus and risk of acute kidney injury after coronary artery bypass grafting. Am Heart J.

[31] Kubal C, Srinivasan AK, Grayson AD, Fabri BM, Chalmers JAC (2005). Effect of risk-adjusted diabetes on mortality and morbidity after coronary artery bypass surgery. Ann Thorac Surg.

[32] Pannu N, Graham M, Klarenbach S, Meyer S, Kieser T, Hemmelgarn B (2016). A new model to predict acute kidney injury requiring renal replacement therapy after cardiac surgery.

[33] Boles Ponto LL, Schoenwald RD (1990). Furosemide (frusemide). A pharmacokinetic/pharmacodynamic review (Part I). Clin Pharmacokinet.

[34] Boles Ponto LL, Schoenwald RD (1990). Furosemide (Frusemide): a pharmacokinetic/pharmacodynamic review (part II). Clin Pharmacokinet.

[35] Brezis M, Agmon Y, Epstein FH (1994). Determinants of intrarenal oxygenation. I. Effects of diuretics. Am J Phys.

[36] Brezis M, Heyman SN, Epstein FH (1994). Determinants of intrarenal oxygenation. II. Hemodynamic effects. Am J Phys.

[37] Arora P, Kolli H, Nainani N, Nader N, Lohr J (2012). Preventable risk factors for acute kidney injury in patients undergoing cardiac surgery. J Cardiothorac Vasc Anesth.

[38] Kidney Disease: Improving Global Outcomes (KDIGO) Acute Kidney Injury Work Group (2012). KDIGO clinical practice guideline for acute kidney injury. Kidney Int Suppl.

[39] Mehta RL, Pascual MT, Soroko S, Chertow GM (2002). Diuretics, mortality, and nonrecovery of renal function in acute renal failure. JAMA.

[40] Ho KM, Sheridan DJ (2006). Meta-analysis of frusemide to prevent or treat acute renal failure. BMJ.

[41] Uchino S, Doig GS, Bellomo R, Morimatsu H, Morgera S, Schetz M, Tan I, Bouman C, Nacedo E, Gibney N, Tolwani A, Ronco C, Kellum JA, Beginning and Ending Supportive Therapy for the Kidney (B.E.S.T. Kidney) Investigators (2004). Diuretics and mortality in acute renal failure. Crit Care Med.

[42] Cantarovich F, Rangoonwala B, Lorenz H, Verho M, Esnault VLM (2004). High-dose furosemide for established ARF: a prospective, randomized, double-blind, placebo-controlled, multicenter trial. Am J Kidney Dis.

[43] Bagshaw SM, Bellomo R, Kellum JA (2008). Oliguria, volume overload, and loop diuretics. Crit Care Med.

[44] Lassnigg A, Donner E, Grubhofer G, Presterl E, Druml W, Hiesmayr M (2000). Lack of renoprotective effects of dopamine and furosemide during cardiac surgery. J Am Soc Nephrol.

[45] Mahesh B, Yim B, Robson D, Pillai R, Ratnatunga C, Pigott D (2008). Does furosemide prevent renal dysfunction in high-risk cardiac surgical patients? Results of a double-blinded prospective randomised trial. Eur J Cardiothorac Surg.

[46] Lal S, Murtagh JG, Pollock AM, Fletcher E, Binnion PF (1969). Acute haemodynamic effects of frusemide in patients with normal and raised left atrial pressures. Br Heart J.

[47] Nelson GI, Ahuja RC, Silke B, Okoli RC, Hussain M, Taylor SH (1983). Haemodynamic effects of frusemide and its influence on repetitive rapid volume loading in acute myocardial infarction. Eur Heart J.

[48] Nelson GIC, Silke B, Forsyth DR, Verma SP, Hussain M, Taylor SH (1983). Hemodynamic comparison of primary venous or arteriolar dilatation and the subsequent effect of furosemide in left ventricular failure after acute myocardial infarction. Am J Cardiol.

[49] Francis GS, Siegel RM, Goldsmith SR, Olivari MT, Levine TBCJ (1985). Acute vasoconstrictor response to intravenous furosemide in patients with chronic congestive heart failure. Ann Intern Med.

[50] Brienza N, Giglio MT, Marucci M, Fiore T (2009). Does perioperative hemodynamic optimization protect renal function in surgical patients? A meta-analytic study. Crit Care Med.

[51] Walsh M, Kurz A, Turan A, Rodseth RN, Cywinski J, Thabane L (2013). Relationship between intraoperative mean arterial pressure and clinical outcomes after noncardiac surgery. Anesthesiology.

[52] Sun LY, Wijeysundera DN, Tait GA, Beattie WS (2015). Association of intraoperative hypotension with acute kidney injury after elective noncardiac surgery. Anesthesiology.

